# Biocontrol Potential of Trichoderma Ghanense and Trichoderma Citrinoviride toward *Pythium aphanidermatum*

**DOI:** 10.3390/jof10040284

**Published:** 2024-04-12

**Authors:** Badriya Khalfan Al-Shuaibi, Elham Ahmed Kazerooni, Dua’a Al-Maqbali, Moza Al-Kharousi, Mohamed N. Al-Yahya’ei, Shah Hussain, Rethinasamy Velazhahan, Abdullah Mohammed Al-Sadi

**Affiliations:** 1 Department of Plant Sciences, College of Agricultural and Marine Sciences, Sultan Qaboos University, P.O. Box 34, Al-Khod 123, Oman; s121416@student.squ.edu.om (B.K.A.-S.); elham.ghasemi.k@gmail.com (E.A.K.); shahpk85@gmail.com (S.H.); velazhahan@squ.edu.om (R.V.); 2 Oman Animal and Plant Genetic Resources Center (Mawarid), Ministry of Higher Education, Research and Innovation, P.O. Box 82, Muscat 112, Oman; duaa.almoqbali@mohe.gov.om (D.A.-M.); moza.alkharousi@moheri.gov.om (M.A.-K.); mohamed.alyahyaei@moheri.gov.om (M.N.A.-Y.)

**Keywords:** antagonistic activity, *Cucumis sativus*, damping-off, endophytes, Oomycetes, plant growth promotion, *Pythium*, *Trichoderma* species

## Abstract

*Pythium*-induced damping-off of cucumber is a major constraint to cucumber production in different parts of the world. Although chemical fungicides are used for managing this disease, they have many drawbacks to the environment. The ability of the antagonistic fungi isolated from the rhizosphere and endosphere of *Dactyloctenium robecchii* and *Moraea sisyrinchium* in the control of soilborne pathogen *Pythium aphanidermatum* was inspected. Native *Trichoderma* isolates, *Trichoderma ghanense* and *Trichoderma citrinoviride*, were isolated from plant stem and soil samples collected from Al-Seeb, Oman. Using a dual culture technique, the antagonistic activity of the fungal isolates against *P. aphanidermatum* was examined in vitro. Among *Trichoderma* isolates, *T. ghanense* was more efficient in restraining the mycelial growth of *P. aphanidermatum*, causing an inhibition percentage of 44.6%. Further, *T. citrinoviride* induced significantly lower cessation of *P. aphanidermatum* mycelial growth (31.3%). Microscopic and electrolyte leakage inspection of the pathogen mycelia depicted extreme morphological malformations in their mycelium, which can be attributed to the antifungal metabolites of antagonists. Greenhouse studies demonstrated the effectivity of *T. ghanense* in controlling *Pythium* damping-off of cucumber plants, where the number of surviving plants was over 90% when the biocontrol agents were used compared to 0 in the control plants. Furthermore, treatment of the plants with the antagonists promoted growth characteristics of plants compared to uninoculated plants. This included improvements in shoot and root lengths, leaf length and width, and dry weight. These findings suggest that *T. ghanense* and *T. citrinoviride* can be developed as alternatives to synthetic chemical fungicides to manage soilborne pathogens of cucumber. This research is also the first to clarify the biocontrol ability of *T. citrinoviride* and *T. ghanense* against cucumber damping-off caused by *P. aphanidermatum*.

## 1. Introduction

Cucumber (*Cucumis sativus* L., family Cucurbitaceae), one of the most important vegetable crops, is grown extensively worldwide [1]. Cucumber fruits are popular around the world for their crispy texture and special flavor. They are an excellent source of vitamins, proteins, minerals, and antioxidants and thus deliver various health benefits to the human body [2,3]. They possess anti-diabetic, lipid-lowering, and antioxidant properties. Moreover, they have Cucurbitacin B and C that prevent tumor growth and guard the liver against inflammation [3,4,5]. The leaves, stems, and roots of the cucumber are commonly employed in Chinese traditional medicine as anti-diarrheal, detoxicant, and anti-gonorrheal medicines [6]. Moreover, the seeds of cucumbers have anti-inflammatory, anti-fever, and antidiabetic effects [7]. Cucumber is one of the three most widely cultivated vegetable crops in Oman. Despite that, this crop is greatly vulnerable to a vast range of diseases leading to economic losses [8].

Plant diseases are believed to be the primary cause of global food production decline, which is estimated to be between 10% and 40%, as they directly contribute to the destruction of natural resources in agriculture [9]. Fungi are the most varied group of plant disease agents and are responsible for one-third of all crop losses each year among soil-borne pathogens, which affect both the natural and production ecosystems [10,11,12]. The dispersion of several phytopathogenic fungi, including *Pythium*, *Fusarium,* and *Rhizoctonia,* owing to the changes initiated in farming, has deleterious impacts on crops of economic importance [13,14,15,16]. Some of these pathogens, such as *Pythium* species, are widely distributed in different parts of the world. They have been shown to result in losses that can reach 75% of cucumbers in greenhouses. The *Pythium* species and other pathogens are particularly difficult to control since each crop may be susceptible to a variety of pathogen species, and they frequently persist in soil for several years through the production of long-lasting spores called oospores [17]. It has been shown that oospores can persist in the soil for up to 4 years in the absence of a host plant [18]. Even when using the normal techniques, they are typically difficult to control.

Even though fungicides are useful for treating a variety of diseases, frequent use of fungicides can have harmful effects on the environment and the survival of helpful rhizosphere microbes [19,20]. Indecisive utilization of fungicides forces pathogens to go through genetic mutation, which ultimately results in the development of fungicide-resistant biotypes [21,22,23]. Fungicide resistance has been reported against several chemical fungicides, especially following their frequent use. *Botrytis cinerea*, for example, a major cause of pre- and post-harvest losses in fruit and vegetable production, can adapt to fungicide treatments through mutations, resulting in resistance and loss of fungicide efficacy [24]. The demand for pesticide-free food among consumers and the rising cost of pesticides have sparked the hunt for alternatives to these goods. Therefore, it is essential to develop accessible and environmentally safe non-chemical techniques for the control of plant diseases. Due to this, artificial fungicides have been replaced by biological control, and utilizing antagonistic microorganisms to manage diseases has resulted in significant success [25,26,27,28].

The efficacy and practicability of non-pathogenic antagonistic fungi and bacteria have been the subject of several studies over the past several decades to commercialize them as biocontrol agents [29,30]. Through conducted studies, a plethora of fungal strains have been created as biocontrol agents, which effectively manage soil-borne diseases of important crops [29,31]. Among the non-pathogenic fungi, *Trichoderma* spp. is a usual saprophytic filamentous fungus that interacts with the soil ecosystem and foliar environment [10]. It plays a role as a biocontrol agent towards varied phytopathogens, causing numerous diseases in crop plants [32,33,34]. It can establish itself in different pathosystems, has the lowest effect on the soil balance, and does not destroy beneficial organisms that help control diseases [23]. It controls phytopathogens by different mechanisms comprising the generation of antifungal compounds, competition for space and nutrients in the rhizosphere, mycoparasitism, and the improvement of host mechanisms for defense [23,35,36].

Numerous *Trichoderma* species have undergone extensive research and are employed for the biological control of a wide range of aerial-borne and soil-borne pathogens, namely *Fusarium oxysporum*, *Rhizoctonia solani*, *Pythium aphanidermatum*, *Alternaria alternata* and *Macrophomina phaseolina* [33,37,38,39,40,41,42,43,44,45,46]. The management capacity varies among *Trichoderma* species and depends on the target disease [34]. The investigation for potential biocontrol agents against cucumber soil-borne disease in Oman resulted in the contemplation of the *Trichoderma* species, which are native to different regions in Oman. *Moraea sisyrinchium* and *Dactyloctenium robecchii* are native plant species to the Arabian Peninsula. *M. sisyrinchium* and *D. robecchii* were considered the most interesting species, especially in terms of their flexibility to grow in various habitats such as hills, mountain floodplains, and saline lands [47,48]. We hypothesized that antagonistic fungi isolated from these plants could enhance pathogen tolerance in cucumber plants under unfavorable conditions. The following were the study goals: (1) isolation of *Trichoderma* species from the rhizosphere and endosphere of *Dactyloctenium robecchii* and *Moraea sisyrinchium*, (2) evaluating the native *Trichoderma* species’ antagonistic effects against *P. aphanidermatum*, and (3) investigating how soil treatment with certain *Trichoderma* species affects cucumber growth and *Pythium* damping-off control.

Research into these areas will help widen the alternative options available for managing *Pythium*-induced diseases in vegetable crops, especially with biocontrol isolates from the environment. In addition, it will help reduce the reliance on chemical fungicides.

## 2. Materials and Methods

### 2.1. Materials Utilized

The study was undertaken in the Plant Pathology Laboratory at Sultan Qaboos University. Chemicals utilized to make various reagents were acquired from Sigma Aldrich, St. Louis, MO, USA. Media were purchased from Oxoid (Thermo Fisher Scientific, Waltham, MA, USA). All reactions were accomplished utilizing sterilized distilled water. The primers for molecular identification were purchased from Macrogen Inc. (Seoul, Republic of Korea). Cucumber seeds were obtained from a local supplier.

### 2.2. Collection Site and Isolation of the Fungal Pathogen

The fungal pathogen, *Pythium aphanidermatum* (isolate no. ON113866), was part of a fungal culture collection of the Department of Plant Sciences (Sultan Qaboos University, Muscat, Oman). It was obtained from our previous studies [49]. On petri dishes containing potato dextrose agar (PDA) medium (Thermo Fisher Scientific, Waltham, MA, USA), the fungal pathogen’s pure culture was transferred and incubated at a temperature of 25 ± 2 °C for further experiments.

### 2.3. Plant Sampling and Isolation of Rhizospheric and Endophytic Fungi

*Dactyloctenium robecchii* (family *Poaceae*) and *Moraea sisyrinchium* (family *Iridaceae*) plants were collected from Al-Seeb (23.6473° N, 58.1458° E), Muscat, Oman. In sterile zipper bags, samples of healthy, symptom-free plants and their rhizospheric soils were transferred to the lab using a mobile refrigeration chamber (4 °C). The identification of plants was carried out at the botany herbarium, located at the Life Science Unit, College of Science, Sultan Qaboos University.

The plant leaves, stems, and roots were all similarly dissected (5 mm) by a sterile scalpel shortly after being thoroughly cleaned with tap water. The dissected fragments were surface sterilized as described by Khalil et al. [50]. The dissected fragments were then briefly immersed in sterile distilled water (60 s), 70% ethanol (60 s), 2.5% sodium hypochlorite (4 min), and 70% ethanol (30 s), and eventually rinsed in sterile distilled water three times. The sterilized fragments were then dried employing sterile filter paper. To validate surface sterilization efficiency, 100 µL of the final rise water was dispersed on a potato dextrose agar (PDA) medium and incubated at 25 ± 2 °C for one week.

The sterilized fragments of the leaves, stems, and roots (5 fragments/plate) were placed on the PDA plate supplemented with rifampicin (10 mg/L) and ampicillin (200 mg/L) to prevent bacterial growth and incubated at 25 ± 2 °C. Every day, plates were checked seeking any evidence of fungal growth. Fungal isolates were sub-cultured onto a PDA medium.

Bulk soils from *D. robecchii* and *M. sisyrinchium* roots were flaked away and the soils that remained attached to the roots were considered rhizospheric soils [51]. The method described by Dey et al. [52] was used to isolate fungi from rhizospheric soil. Corresponding to the rhizospheric soil, root specimens were agitated at high speed for 90 s to collect rhizospheric soil. Thereafter, 1 g of collected soil was taken and diluted in 10 mL of sterile distilled water and labeled as stock solution. Subsequently, the serial dilution method was used to dilute the stock solution and minimize the fungi in the soil in each dilution. Eventually, 0.1 mL of the solution in each soil dilution was added to the prepared PDA medium, which was then incubated for seven days at 25 °C.

For further examination, pure cultures of the acquired fungus were commonly maintained on PDA slants at 4 °C and categorized according to their cultural appearance. Additionally, the isolated fungi were identified using morphological and molecular analysis.

### 2.4. Characterization of Fungal Isolates

#### DNA Extraction, Amplification, and Sequencing

The isolated fungi were identified at the genus level based on cultural and morphological characteristics, and microscopical attributes as stated previously [53,54]. For molecular identification, genomic DNA was extracted from fungal isolates following the protocol of Al-Sadi et al. [55]. The extracted DNA’s quality and quantity were determined by a NanoDrop^TM^ 2000 spectrophotometer (Thermo Fisher Scientific, Waltham, MA, USA). DNA was preserved at −20 °C until use. Utilizing the primer combination ITS1 (5′-TCC GTA GGT GAA CCT GCG G-3′) and ITS4 (5′-TCCTCCGCTTATTGATATGC-3′); EF1-526F (GTC GTY GTY ATY GGH CAY GT) and EF-jR (GCR TGY TCN CGR GTY TGN CCR TC), we amplified the nuclear ribosomal DNA’s internal transcribed spacer (ITS1-5.8S-ITS2 = ITS) and a portion of the translation elongation factor 1 alpha (EF1α) region, respectively [56]. Polymerase chain reactions were performed using the PuRe Taq Ready-To-Go™ PCR beads (Cytiva, Marlborough, MA, USA), with 1 µL of each primer (10 µM/µL), DNA (1 µL), and 25 µL sterile distilled water (25 µL) following the protocol of Lakhani et al. [57]. On a 1% agarose gel (140 min, 80 V, 400 mA), PCR-amplified products were evaluated for their supposed size and visualized with ethidium bromide under UV illumination. PCR-amplified products were purified and sequenced with the same primers from Macrogen Inc. (Seoul, Republic of Korea). The resulting sequences were deposited in GenBank. The type sequences of closely related species of *Trichoderma* were selected for phylogenetic analysis. Sequences were aligned using the MAFFT algorithm [58]. The maximum likelihood phylogenetic method was used for phylogenetic analysis using RAxMLHPC2 v. 8.2.4. The resulting phylogenetic tree was visualized in FigTree 1.4.2 [59] and annotated using Adobe Illustrator CC2019.

### 2.5. Efficacy of Rhizospheric and Endophytic Fungi as Biocontrol Agents against P. aphanidermatum

#### 2.5.1. In Vitro Evaluation of the Fungal Isolates’ Antifungal Activity towards Fungal Pathogens

Two *Trichoderma* isolates were chosen among the obtained rhizospheric and endophytic fungal isolates and their antagonistic effects against *P. aphanidermatum* were examined via a dual culture approach [60]. The PDA medium-filled petri plates with a 90 mm diameter were used for this test. The pathogen agar–mycelium cylinder (*P. aphanidermatum*, 6 mm diameter), cut from the edge of an actively growing fungal colony, was placed on the edge of the plate. The non-pathogen agar–mycelium disc (6 mm diameter) was inoculated on the same day and placed on the opposite edge. Three replicates were arranged for each isolate and plates inoculated only with pathogen were considered as a control. After the inoculation, the plates were kept at 25 °C until the leading edge of the fungus on the control plate reached the edge of the plate. By comparing the percentage of mycelium growth inhibition to the control, the antagonistic activity was shown [61]. A scanning electron microscope (SEM; JEOL JSM-5600, Tokyo, Japan) was also used to see how the selected fungal isolates affected the hyphal morphology of *P. aphanidermatum*. A protocol defined by Heckman et al. [62] was pursued for the SEM sample preparation.

#### 2.5.2. Effect of Trichoderma Isolates on Extracellular Conductivity of *P. aphanidermatum*

The impact of *Trichoderma* isolates on electrolyte leakage of *P. aphanidermatum* was studied as described previously [63,64]. 5 mm mycelial discs of *Trichoderma* isolates (3-day-old, previously grown on PDA) were transferred into 50 mL sterile conical flasks containing 25 mL potato dextrose broth (PDB, Sigma Aldrich, MO, USA) and, subsequently, incubated at 27 °C in a shaker for 7 days. The resulting suspension was centrifuged at 10,000 rpm for 10 min to separate the fungal mycelium and culture filtrate. The culture filtrate was then filtered using Minisart filters with 0.2 μm pore size for the purity of the culture filtrates. The obtained supernatant was preserved at 4 °C. Moreover, *Pythium* isolate was grown in potato dextrose broth (PDB) for 3 days in an incubator at 27 °C. After that, sterile filter paper was used to collect the *Pythium* mycelium, which was then thoroughly cleaned with sterile double-distilled water. Following that, 3 mg of the collected mycelium of *Pythium* was added to the glass vial containing the *Trichoderma* culture supernatant (20 mL). Eventually, the mycelial suspension was centrifuged at 10,000 rpm for 10 min to collect the supernatant, first immediately after the addition of mycelium (0 h), second after 5 h, and third after 24 h of treatment. A conductivity meter (Mettler Toledo FiveGo™, Herisau, Switzerland) was used to measure conductivity.

### 2.6. Biocontrol Potential of Trichoderma Isolates against P. aphanidermatum on Cucumber in Pots

#### Fungal Inoculum Preparation, Experimental Design, and Plant Growth Condition

A pot experiment was outlined using pots (15 cm × 15 cm) containing autoclaved-sterilized potting mixture (300 g/pot) (Bulrush Horticulture, Ireland, UK) to test the efficacy of *Trichoderma* in promoting plant growth. The potting soil was infested with *P. aphanidermatum* grown on millet grains at 1% (*w*/*w*). 10 mL spore suspensions (1 × 10^8^ spore/mL) of the selected *Trichoderma* spp. isolates were added to the pots, followed by watering the pots for 3 days before sowing [65]. After disinfecting the cucumber seeds (*Cucumis sativus* cv. Azza) (AgriMax Company, Barka, Oman) in 1.5% sodium hypochlorite for two minutes and 70% ethanol for one minute, they underwent three rinses in sterile distilled water. The disinfected seeds were then planted in pots (5 seeds/pot) with 5 replicates for each treatment. The growth-promoting experiment comprised 4 treatments: non-infested soil (control), soil treated with *P. aphanidermatum* only, soil treated with *Trichoderma ghanense* only, and soil treated with *Trichoderma citrinoviride* only. The experimental design for the seedling survival experiment is described in Table 1. The pots were incubated in a greenhouse and grown under natural daylight, 25 °C/20 °C (day/night) temperature and 80% relative humidity. Seed germination and disease percentage of the seedlings were examined after 10 days.

Multiple plant growth characteristics were assessed to ascertain the consequences of each treatment on the plant growth attributes. These metrics, which were measured after ten days, included shoot length, root length, leaf area (length/width), and total plant fresh weight.

### 2.7. Statistical Analysis

The obtained data (stated as the mean ± SD) were exposed to statistical analysis. R software (version 4.0.3) and Microsoft Excel 2021 were used to perform statistical analysis. At a 5% probability level (*p* < 0.05), the least significant difference test (LSD) and analysis of variance were used to compare the means. The graphs were created with the aid of the GraphPad Prism program (version 6.01, San Diego, CA, USA).

## 3. Results

### 3.1. Identification of the Antagonistic Fungi

**Figure 1:**
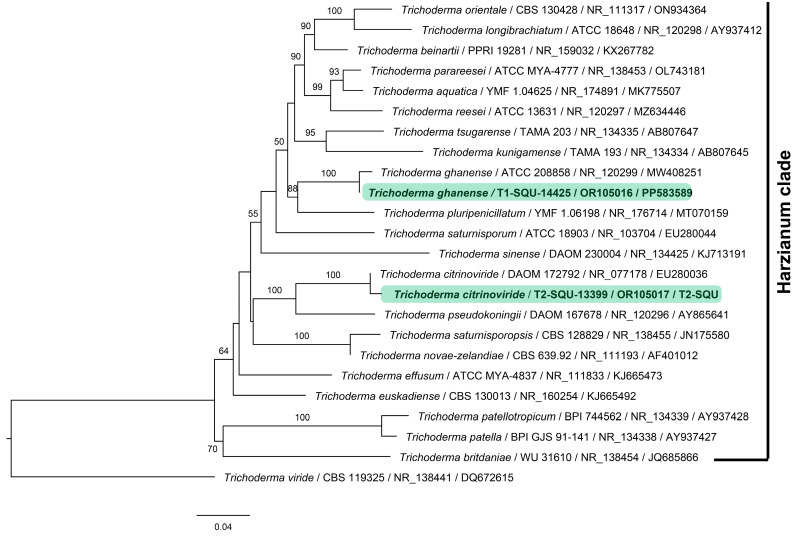
Maximum likelihood phylogenetic tree based on combined ITS–EF1α dataset obtained from 24 type specimens of *Trichoderma* species of Harzianum clade. Each leaf in the tree is presented as species name followed by isolate or strain number, ITS accession, and EF1α accession, separated by a/line, respectively. The representative species *Trichoderma citrinoviride* (T2-SQU-13399) and *T. ghanense* (T1-SQU-14425) are highlighted in green; bootstrap support values above 50% are consistently significant and are shown.

The fungal isolates were identified as *Trichoderma ghanense* (T1) and *Trichoderma citrinoviride* (T2) using phylogenetic analysis of the combined ITS–EF1α sequence data. Both these species are members of the Harzianum clade. The phylogenetic tree depicted in Figure 1 consists of types of sequences of *Trichoderma* species, presented as species name, followed by isolate or strain number, ITS accession, and EF1α accession, respectively (Table 2). The Omani isolates are highlighted in green.

### 3.2. Antifungal Activity of the Trichoderma Isolates against Fungal Pathogen (P. aphanidermatum)

#### 3.2.1. Dual Culture and Scanning Electron Microscope Assays

**Figure 2:**
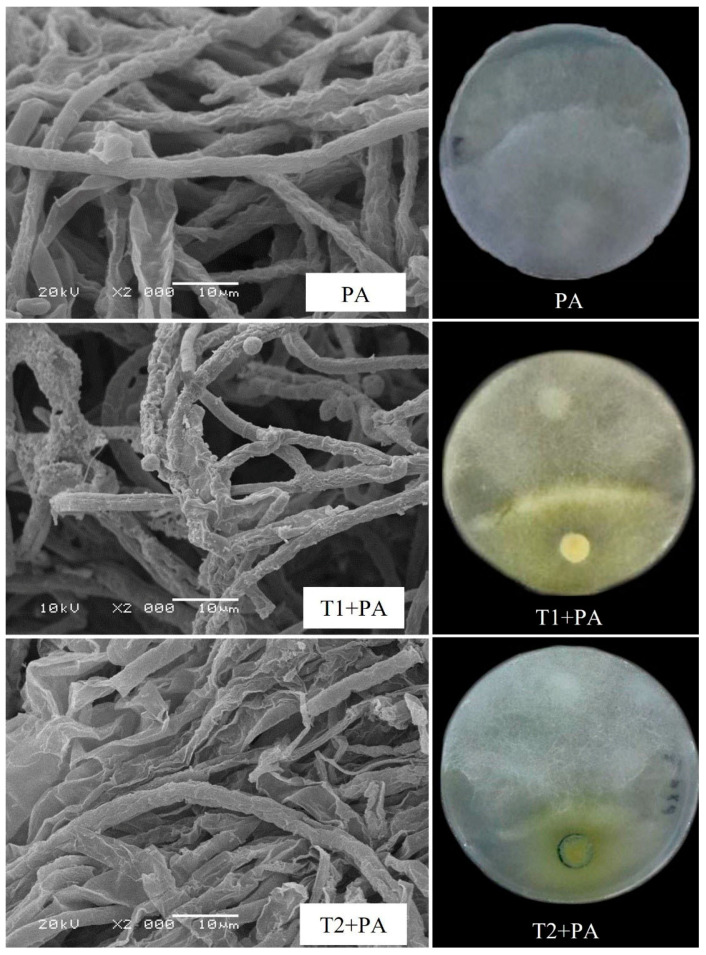
Effect of *Trichoderma ghanense* (T1) and *Trichoderma citrinoviride* (T2) on *Pythium aphanidermatum* (PA) mycelial morphology depicted using dual culture assay and scanning electron microscope (SEM). Distorted mycelial structure, wrinkled, ruptured, or shrunken patterns in T1 + PA and T2 + PA plates. Typical hypha patterns were visible in the control (PA).

**Figure 3:**
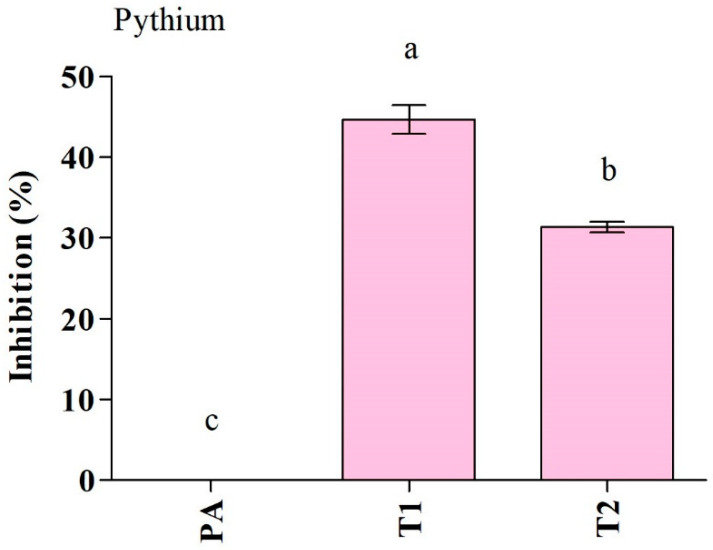
Antagonistic activity of *Trichoderma ghanense* (T1) and *Trichoderma citrinoviride* (T2) and their effect on the inhibition of *Pythium aphanidermatum* (PA) growth. Values show the means ± standard error (*n* = 3) and significant differences at *p* < 0.05 are indicated by different lowercase letters above the columns.

The *Trichoderma* isolates were tested for their ability to suppress *P. aphanidermatum* growth in vitro (Figure 2, Table 2). Among the *Trichoderma* isolates, *T. ghanense* (T1) was found to be more efficient in restraining the mycelial growth of *P. aphanidermatum* compared to *T. citrinoviride* (T2). *T. ghanense* caused inhibition percentages of 44.6% for *P. aphanidermatum* (Figure 3). However, *T. citrinoviride* (T2) caused a considerably lower suppression of *P. aphanidermatum* mycelial growth (31.3%). Scanning electron microscopy (SEM) was used to confirm that *T. ghanense* (T1) and *T. citrinoviride* (T2) isolates had an impact on the hyphal morphology of *P. aphanidermatum* (Figure 2). The scanning electron microscope revealed that *Trichoderma* isolates T1 and T2 significantly altered the hyphal morphology of *P. aphanidermatum* (Figure 2). When compared to the control, the majority of the observed hyphal patterns were wrinkled and ruptured in both treatments.

#### 3.2.2. Extracellular Conductivity

**Figure 4:**
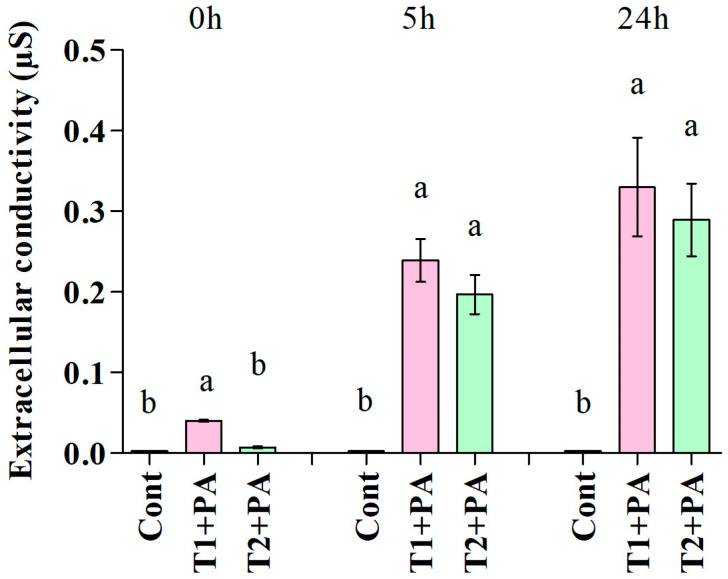
Effect of antifungal metabolites (*Trichoderma ghanense*, T1; *Trichoderma citrinoviride*, T2) on extracellular conductivity in the suspension of the pathogen (*Pythium aphanidermatum*, PA) after 0, 5, and 24 h of treatment. Values show the means ± standard error (*n* = 3) and significant differences at *p* < 0.05 are indicated by different lowercase letters above the columns.

Exposure of *P. aphanidermatum* to the culture filtrate of *T. ghanense* and *T. citrinoviride* for 5 and 24 h led to enhanced levels of extracellular conductivity in comparison to the control, which depicted cellular electrolyte leakage from *P. aphanidermatum* owing to losing cell wall/cell membrane integrity. Among the antagonists, *T. ghanense* depicted the maximum discharge of electrolytes from the fungal mycelium (Figure 4).

### 3.3. Effect of Trichoderma Isolates on Cucumber Growth and Damping-Off Disease

**Figure 5:**
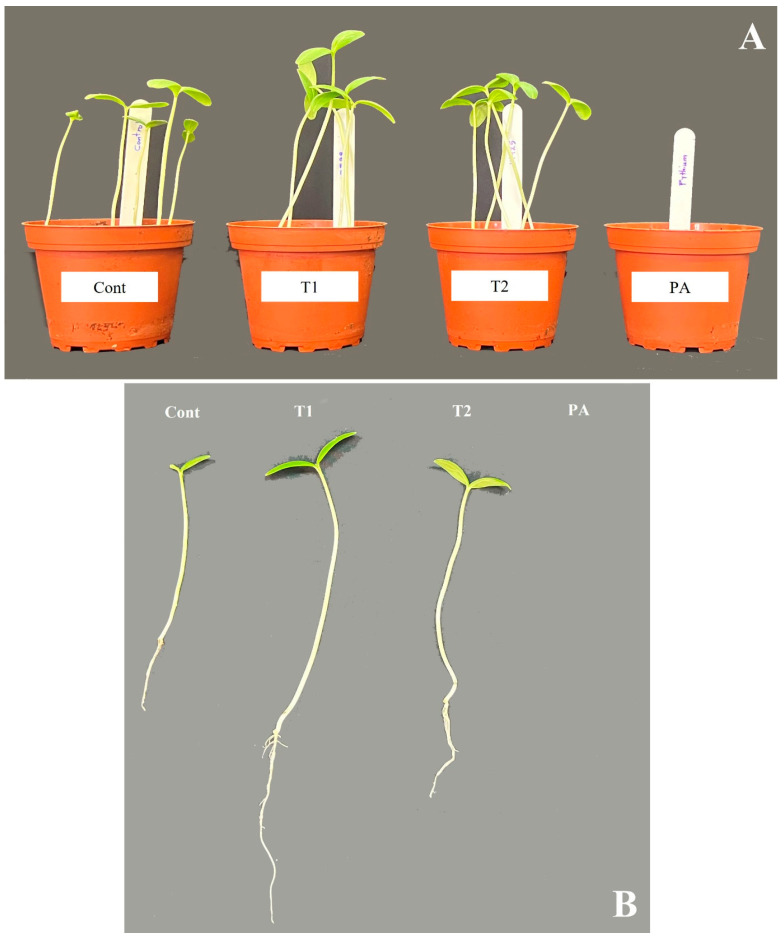
Growth-promoting impact of *Trichoderma* isolates on cucumber plants treated with fungal pathogens over ten days (**A**,**B**). Treatment: control (Cont), *Trichoderma ghanense* (T1), *Trichoderma citrinoviride* (T2), *Pythium aphanidermatum* (PA).

**Figure 6:**
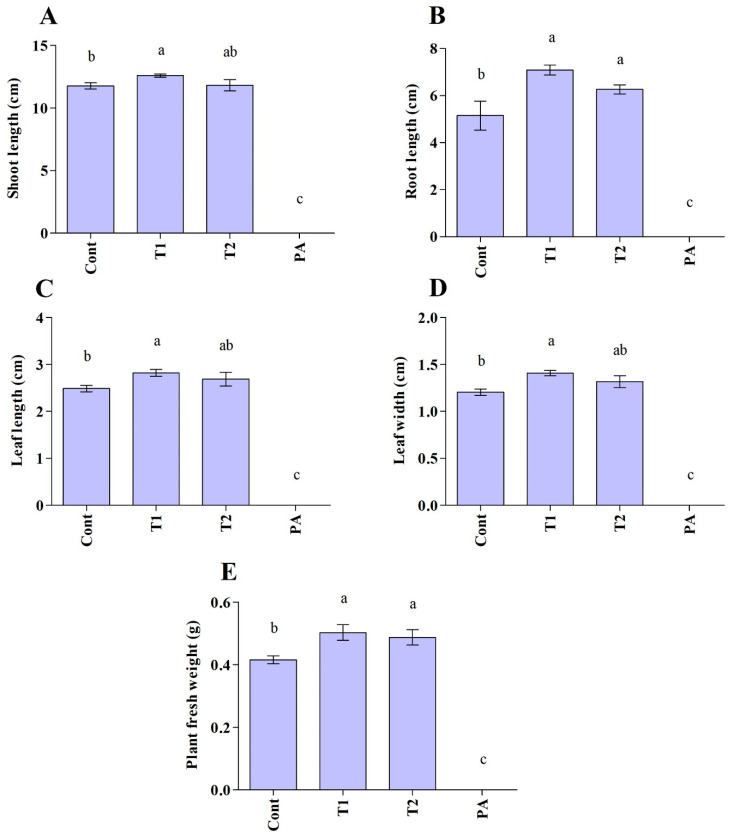
Plant growth-promoting effect of *Trichoderma* isolates on the cucumber plants under normal and disease conditions after ten days (**A**–**E**). Treatment groups: control (Cont), *Trichoderma ghanense* (T1), *Trichoderma citrinoviride* (T2), *Pythium aphanidermatum* (PA). Values show the means ± standard error (*n* = 5) and significant differences at *p* < 0.05 are indicated by different lowercase letters above the columns.

**Figure 7:**
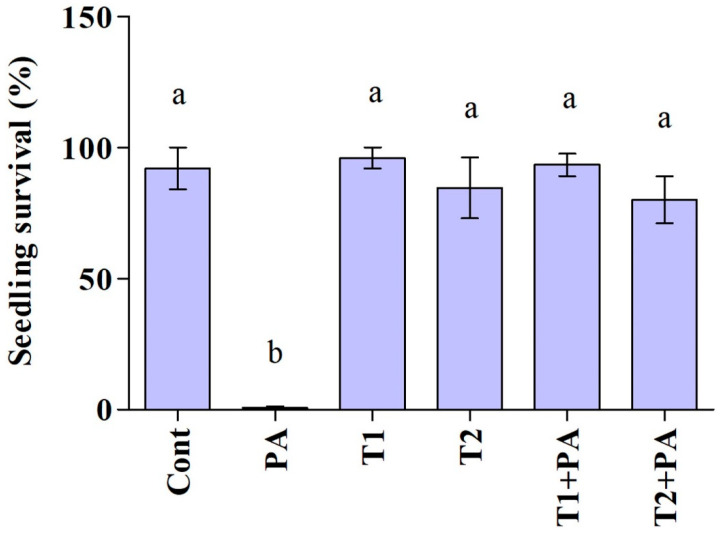
Biocontrol potential (survival percentage) of the *Trichoderma* isolates on the *Pythium aphanidermatum* causal agent of cucumber damping-off in pot experiment over ten days. Treatment groups: control (Cont), *Pythium aphanidermatum* (PA), *Trichoderma ghanense* (T1), *Trichoderma citrinoviride* (T2), *Trichoderma ghanense* (T1) + *Pythium aphanidermatum* (PA), and *Trichoderma citrinoviride* (T2) + *Pythium aphanidermatum* (PA). Values show the means ± standard error (*n* = 5) and significant differences at *p* < 0.05 are indicated by different lowercase letters above the columns.

The impact of the *Trichoderma* isolates on cucumber seedlings was investigated (Figure 5 and Figure 6). The unfavorable consequences of *P. aphanidermatum* invasion resulted in the mitigation of seed germination and growth parameters; namely, shoot length, root length, leaf area (length/width), and total fresh weight of the cucumber plants, as compared to non-diseased, un-treated cucumber plants. However, disease severity was considerably decreased in antagonist-treated plants. *Trichoderma*-treated cucumber plants emerged higher and stronger than plants treated with pathogen only, and the seeds had 100% germination in control and *Trichoderma*-treated plants. In comparison to plants exposed to *P. aphanidermatum*, *Trichoderma* treatment increased shoot length, root length, leaf area (length/width), and total fresh weight of the inoculated plants. When compared to similar untreated sick plants, the shoot length increased by 100% in the *T. ghanense* treatment group and by 100% in the *T. citrinoviride* treatment group (*p* < 0.05). Similarly, in the *T. ghanense* and *T. citrinoviride* treatment groups, compared to the diseased plants, the overall plant fresh weight of *Trichoderma*-treated plants increased by 100% (Figure 6). As depicted in Figure 7, the survival percentage of diseased plants inoculated by *T. ghanense* and *T. citrinoviride* improved by 98.92% and 98.75%, respectively, in contrast to the untreated diseased plants.

## 4. Discussion

Plant diseases caused by pathogenic microorganisms induce enormous losses that are reckoned to range between 13 and 22% [66,67]. Given that chemical control has many side effects [68], the use of biological control is promising [69]. Cucumber damping-off is an extremely severe disease resulting in serious losses to cucumber production. Managing cucumber damping-off through the use of chemical fungicides has both benefits and drawbacks. Controlling damping-off via biological methods offers a safe alternative [70]. *Trichoderma* is a biocontrol fungus that is found all over the world. *Trichoderma* has enormous practical value and potential in the realm of biological plant disease control [9]. *Trichoderma* has been studied for its ability to control plant diseases all over the world. *T. viride* and *T. harzianum* have been shown to be effective in inhibiting around 30 plant pathogenic fungi, including *Fusarium*, *Pythium* and *Rhizoctonia*. *Rhizoctonia solani*, *Pythium aphanidermatum*, *Sclerotinia sclerotiorum*, and *Colletotrichum* spp., which are all controlled by the *Trichoderma* spp. [71]. A study by Paulitz et al. [72] demonstrated the efficiency of *T. harzianum* in reducing *Pythium* damping-off of cucumbers in greenhouse experiments. *Trichoderma* is a good substitute for chemical pesticides that could be more trustworthy and ecologically safe, along with economically endurable [73]. The current study identified *T. ghanense* and *T. citrinoviride* as endophytic and rhizospheric fungi in *D. robecchii* and *M. sisyrinchium*, respectively. *T. ghanense* and *T. citrinoviride* were identified at the species level based on sequences of the ITS, which was useful in distinguishing these species from other *Trichoderma* species. These two *Trichoderma* species were not isolated previously from *D. robecchii* and *M. sisyrinchium*.

In this study, *T. ghanense* and *T. citrinoviride* reduced the growth of *P. aphanidermatum in vitro*. A study conducted by Park et al. [74] depicted the effective mycoparasitic activity of *T. citrinoviride* via dual culture assays against ginseng pathogens, including *Rhizoctonia solani*, *Botrytis cinerea*, and *Pythium* spp. Their results showed that the cell wall degrading enzyme, endoglucanase, contributed to the antagonistic activity of *T. citrinoviride* toward pathogens. Moreover, a study by Khadka et al. [75] revealed reduced mycelial growth of *Rhizoctonia solani* by *T. ghanense*.

Mycoparasitism is a key biological control mechanism for *Trichoderma*. *Trichoderma* can parasitize 18 different *Pythium*, *Phytophthora*, *Rhizoctonia*, and *Peronospora* genera. They can infiltrate or injure the mycelium, causing pathogen cells to enlarge, distort, and rupture [76,77]. In addition to dual culture assays, scanning electron microscopy observations of pathogenic fungal mycelia from the border of inhibition zones exhibited serious structural modifications in mycelial structure, which demonstrated that *Trichoderma* metabolites possibly attack the cell wall/cell membrane. According to Halifu et al. [78], *Trichoderma* spp. can permeate into a pathogen body by producing enzymes such as chitinase, cellulase, and xylanase, and grow thoroughly within mycelium via demolishing the cell wall.

The use of culture filtrates from *T. ghanense* and *T. citrinoviride* led to the leakage of electrolytes from the *P. aphanidermatum* mycelium. Extracellular conductivity of *T. ghanense* and *T. citrinoviride* supernatants that were treated with *P. aphanidermatum* mycelium was escalated with the proceeding of time in comparison to 0 min. Antifungal compounds mediated the action. Under in vivo conditions, the interaction’s influence was noticeable for diminished fungal diseases. Zhang et al. [79] indicated that the culture filtrate of *Trichoderma* spp. negatively influenced the growth of *Sclerotinia sclerotiorum*.

*Trichoderma* is a saprophytic fungus with rapid mycelial development and high environmental adaptation. It can prevent pathogenic fungi from invading plant roots. It can also rapidly compete for the nutrients essential for disease fungal growth, resulting in nutritional shortage and preventing pathogen fungi growth and reproduction [80,81]. In the bioassay experiment, it was found that *T. ghanense* and *T. citrinoviride* were very efficient in diminishing the severity of the damping-off disease of cucumber as induced by *P. aphanidermatum*. With or without *Pythium* infection, *Trichoderma* inoculation led to higher growth and better health in the cucumber plants. Compared to plants without *Trichoderma*, inoculated plants maintained greater height, root length, leaf area, and plant/root weight. In addition, *Trichoderma* isolates suppressed *Pythium*-induced damping-off of cucumber. Various mechanisms have been suggested to clarify the beneficial effects of *Trichoderma* spp. on plants. One feature is connected to the broad range of metabolites that they generate. It has been confirmed that these metabolites not only interfere with the growth of the pathogen but also enhance disease endurance by inducing the defense responses in the host plant. Moreover, these metabolites have been shown to improve the growth of plants, which enables plants to tolerate infection by compensating for the lost tissues due to pathogen invasion [78,82,83].

## 5. Conclusions

The study focused on investigating the suppressive effects of *Trichoderma* species against *Pythium aphanidermatum*. Along with enhancing cucumber growth, *Trichoderma ghanense* and *T. citrinoviride* were found to suppress *Pythium* damping-off in cucumber. To our knowledge, this study is the first to investigate the suppressive effects of *T. ghanense* and *T. citrinoviride* on the damping-off disease of cucumber caused by *P. aphanidermatum*. The results of this study indicate that *T. ghanense* and *T. citrinoviride* are beneficial for *C. sativus* under disease attack and can be used in the management of *Pythium* diseases. Overall, employing these *Trichoderma* species to manage *Pythium* damping-off is not only associated with retarding fungal growth, but it can also reduce the usage of fungicides, and be used as potential biocontrol agents in organic farms.

## Figures and Tables

**Table 1:** Experimental work plan for the seedling survival experiment.

Symbol	Treatment
Cont	treated with sterile distilled water
T1	treated with *Trichoderma ghanense*
T2	treated with *Trichoderma citrinoviride*
PA	treated with *Pythium aphanidermatum*
T1 + PA	treated with *Trichoderma ghanense* + *Pythium aphanidermatum*
T2 + PA	treated with *Trichoderma citrinoviride* + *Pythium aphanidermatum*

**Table 2:** Identification of potential strains of *Trichoderma* sp. associated with *Dactyloctenium robecchii* and *Moraea sisyrinchium* and its antagonistic activity (+indicates a positive response). EF1α sequences were submitted to GenBank.

Sample Code	Fungal Species	Isolated Host	Source of Isolation	GenBank Accession No.		Location	Antagonistic Activity
				ITS	EF1α		*Pythium aphanidermatum*
T1	*Trichoderma ghanense*	*Dactyloctenium robecchii*	stem	OR105016	PP583589	Al-Seeb	+
T2	*Trichoderma citrinoviride*	*Moraea sisyrinchium*	soil	OR105017	T2-SQU *	Al-Seeb	+

* The EF1α sequence is availbale upon request (isolate # T2-SQU).

## Data Availability

The ITS sequence data generated during the current study are available in GenBank under the accession number OR105016 and OR105017.
